# Does spatialized audio enhance the creation of mental representations?

**DOI:** 10.3389/fnins.2025.1660373

**Published:** 2025-10-16

**Authors:** Shehzaib Shafique, Silvia Zanchi, Walter Setti, Monica Gori, Lorenzo Picinali

**Affiliations:** ^1^Unit for Visually Impaired People (U-VIP), Italian Institute of Technology, Genova, Italy; ^2^Audio Experience Design (AXD), Imperial College London, London, United Kingdom

**Keywords:** binaural, spatialized audio feedback, LEGO, mental representation, blind navigation, spatial navigation, perception

## Abstract

Navigating unfamiliar environments without vision is a considerable challenge for blind individuals, as it requires constructing accurate cognitive maps. Binaural audio feedback, which delivers spatialized auditory cues, has been proposed as a means of enhancing spatial navigation by leveraging the auditory system's natural ability to localize sounds in three dimensions. This study investigated whether binaural audio feedback offers measurable advantages over non-spatialized feedback in supporting spatial perception and mental representation. Fourteen participants, seven blind individuals and seven blindfolded sighted individuals, explored controlled environments under both feedback conditions and reconstructed the layouts using LEGO models. Performance was evaluated through spatial correlation analysis and distance accuracy measures. Results revealed no significant differences between binaural and non-spatialized conditions for either group. These findings indicate that spatialization of descriptive audio alone may not be sufficient to enhance spatial representations, suggesting that factors such as prior training, task design, and integration with other sensory cues may be critical for unlocking the full potential of binaural audio in assistive navigation.

## 1 Introduction

Navigating unfamiliar environments without vision is highly challenging for blind individuals, as constructing cognitive maps is often a lengthy and complex process. Auditory information, however, can provide an effective means of compensating for the absence of vision by conveying cues about distance, direction, and environmental structure (Ohuchi et al., 2006). In normal-hearing populations, spatial hearing relies on interaural time and level differences, reverberation, and spectral shaping, which together support both egocentric and allocentric representations of space (Kolarik et al., 2016; Town et al., 2017). These auditory spatial skills are not consistent across all populations: hearing-impaired individuals frequently show reduced accuracy in orientation and navigation tasks, emphasizing the essential role of auditory input for spatial cognition (Avci and Cicek Cinar, 2024). In contrast, blind individuals often exploit auditory cues more extensively, and in some cases demonstrate preserved or enhanced spatial memory skills relative to sighted individuals (Setti et al., 2018). Taken together, this evidence highlights the central role of auditory input in spatial abilities across diverse groups and provides a foundation for examining whether spatialized audio can further support cognitive mapping in visually impaired individuals.

Navigation through space is crucial for individuals with blindness or visual impairment to maintain independence and travel safely. Traditional navigation aids such as canes and guide dogs provide valuable assistance but often lack the ability to convey precise geographical information. Recent advances in acoustic technology have introduced auditory-based feedback systems as potential solutions. Among these, binaural audio feedback has been proposed as a promising tool for enhancing navigation in three-dimensional environments (Chen et al., 2020; Nguyen et al., 2023). Binaural feedback leverages spatial hearing by incorporating interaural time and level differences to simulate realistic 3D soundscapes, allowing users to perceive objects and landmarks as if they occupy specific positions in space (Sunder, 2021). In contrast, non-spatialized audio feedback refers to monophonic sound, where the audio signal is collapsed into a single channel. While it can still provide semantic information about object identity or relative arrangement, it lacks spatial separation and conveys no sense of depth or direction. Comparing binaural and non-spatialized conditions therefore allows for assessing the specific contribution of spatialized auditory cues to navigation and spatial cognition.

Research has demonstrated that blind individuals develop superior auditory abilities. For instance, Doucet et al. (2005) concluded that blind subjects outperform others in azimuthal localization, showing a greater ability to process spectral indications even if spectrum degradations occur. Röder and Rösler (2003) pointed out that blind individuals also show better recollection of noises, further confirming their advanced auditory processing. Additionally, in the absence of vision, kinesthetic sensations serve as a useful substitute for making mental maps of the environment (Nakaya, 1997).

Binaural auditory feedback is hypothesized to leverage the natural three-dimensional localization ability of the human auditory system. This feedback, delivered through headphones that provide spatial information by simulating the presence of objects and landmarks, has been suggested as a means for blind individuals to create mental maps and increase the accuracy of their navigation. Binaural audio cues have been implemented in assistive technologies designed to help blind individuals navigate complex environments (Hoon et al., 2015). Some studies suggest that this method enhances independence and spatial awareness (Afonso-Jaco and Katz, 2022; Finocchietti et al., 2023), though its effectiveness in constructing precise spatial representations remains an open question.

Moreover, studies indicate that early blind individuals develop superior auditory spatial abilities compared to sighted individuals (Voss et al., 2004). Even in non-spatialized audio feedback environments, they are more adept at utilizing spectral cues and pinpointing individual sound sources (Finocchietti et al., 2023). These findings suggest that binaural auditory feedback could potentially facilitate more efficient spatial navigation by compensating for the lack of visual cues, but its advantages over non-spatialized audio in practical settings remain debated. The efficiency of binaural audio feedback has been further explored through integration with other technologies, such as augmented reality and real-time locating systems, which enhance users' overall navigation experience by providing dynamic, context-sensitive aural cues (Ferrand et al., 2018).

The present study aimed to evaluate whether binaural auditory feedback provides a measurable advantage over non-spatialized feedback in supporting spatial representation. Although previous research has suggested potential benefits of binaural cues for spatial awareness and navigation (Afonso-Jaco and Katz, 2022; Finocchietti et al., 2023), direct comparisons between binaural and non-spatialized conditions in blind individuals remain scarce. Our primary hypothesis was that participants would demonstrate higher accuracy in reconstructing spatial layouts when using binaural feedback compared to non-spatialized feedback, as the additional spatial cues were expected to enhance localization and spatial awareness. A secondary hypothesis was that blind participants would perform as well as, or better than, blindfolded sighted participants, reflecting their extensive reliance on auditory input in everyday navigation. Finally, we considered an alternative possibility: that binaural feedback may not confer significant advantages without prior training or adaptation, which would challenge the assumption that spatialized sound alone is sufficient to improve spatial cognition.

## 2 Methodology

### 2.1 Participants

Because the recruitment of blind individuals is often constrained by participant availability, the sample size in this study was determined based on feasibility. A total of seven blind participants were recruited (4 males, 3 females), and to allow balanced group comparisons, seven blindfolded sighted participants were also included (5 males, 2 females). The blind group had a mean age of 38.4 ± 4.5 years (range: 32–45 years), while the blindfolded sighted group had a mean age of 29.4 ± 3.2 years (range: 25–34 years). Although the groups were not formally age-matched, both fell within a comparable adult age range, and no participant reported age-related cognitive decline.

Participants were recruited between June 2024 and December 2024. Blind individuals were drawn from the Italian Institute of Technology (IIT) database, while blindfolded sighted individuals were recruited separately. The inclusion of both groups allowed us to assess whether the effects of spatialized audio are specific to blindness or reflect general auditory processing mechanisms. No standardized cognitive screening tool (e.g., MoCA) was administered, as all participants were drawn from established research databases and showed intact cognitive functioning during instructions and practice sessions. Hearing status was assessed through self-report: none of the participants reported hearing impairments or use of hearing aids, and all confirmed normal hearing sufficient for everyday communication.

All participants provided written informed consent before their involvement in the study, in accordance with ethical standards outlined in the Declaration of Helsinki. The study protocol was thoroughly reviewed and approved by the local health service ethics committee (*Comitato Etico, ASL 3, Genoa, Italy*), ensuring compliance with ethical and legal research guidelines. The duration of each session varied based on the individual participant's abilities and engagement level, but, on average, each session lasted ~ 40 min.

### 2.2 Experimental setup

**Figure 1:**
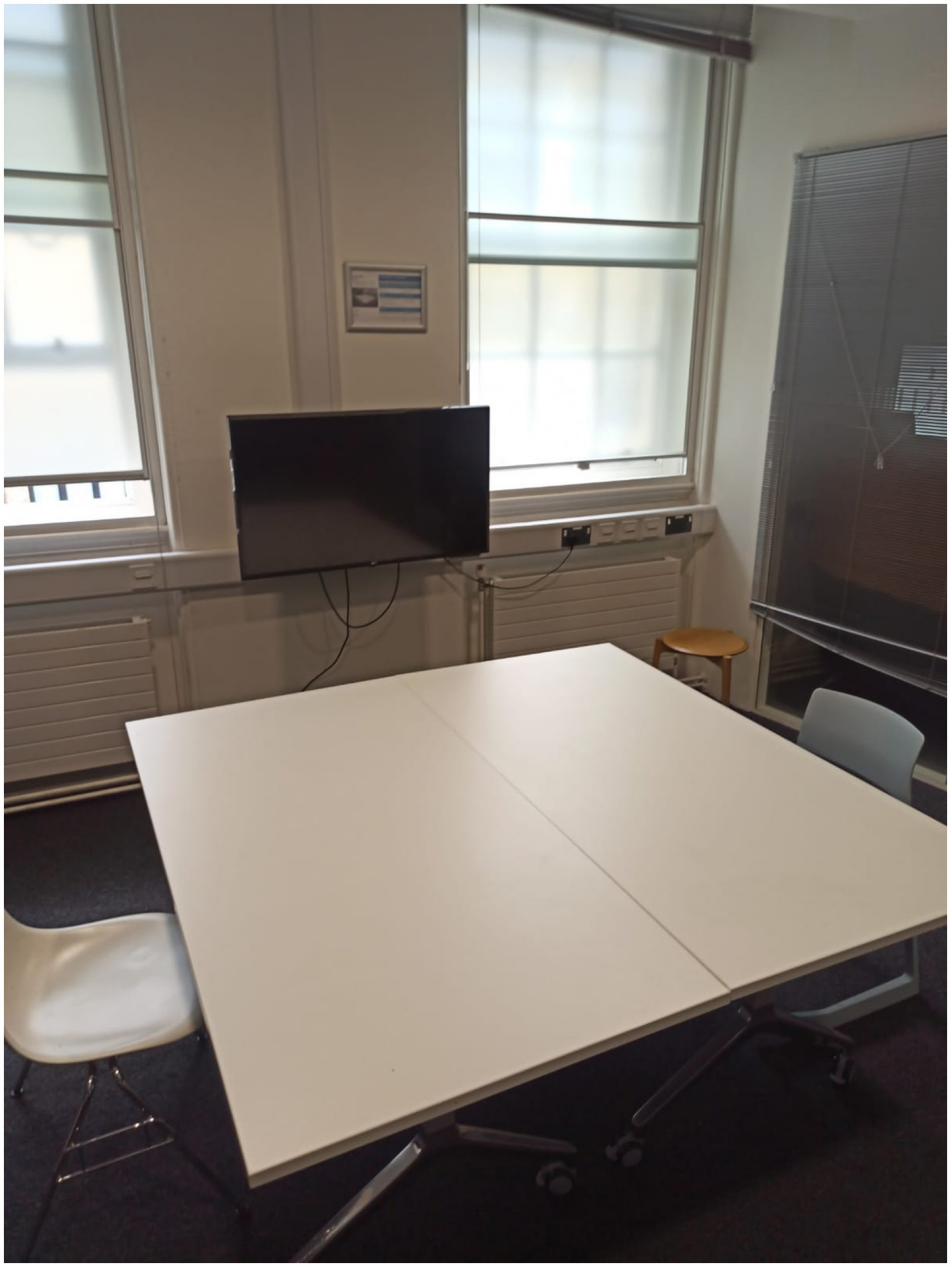
One of the two room layouts used for blindfolded sighted participants, containing two chairs, one table, one wooden stool, and a TV screen. A second layout with the same set of objects arranged differently was also employed. Both the feedback condition (binaural vs. non-spatialized) and the room layout were randomized across participants to minimize learning or carryover effects.

**Figure 2:**
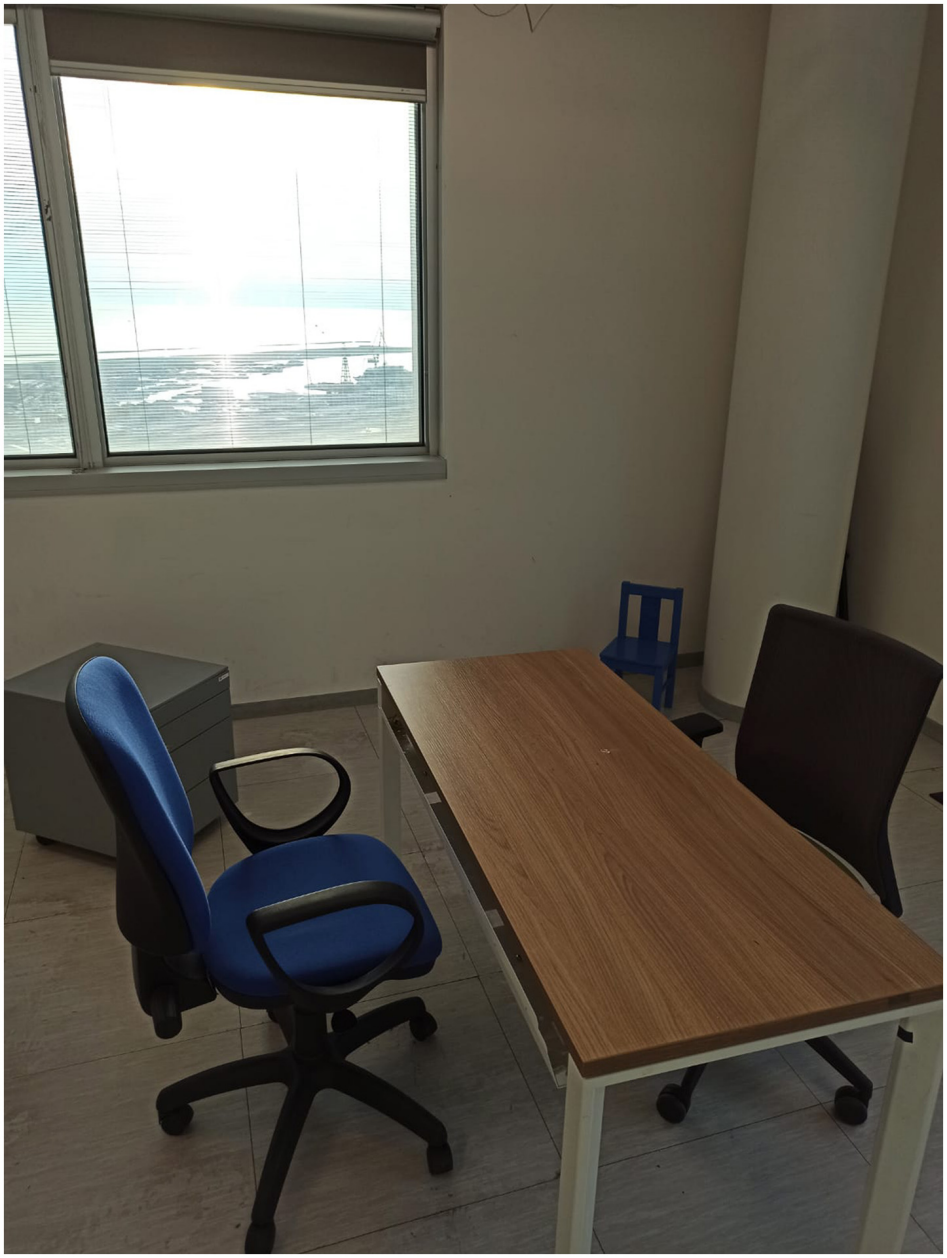
One of the two room layouts used for blind participants, containing one table, two large chairs, one small chair, and a drawer box. A second layout with the same set of objects arranged differently was also employed. Both the feedback condition (binaural vs. non-spatialized) and the room layout were randomized across participants to minimize learning or carryover effects.

For the experimental setup, two rooms were prepared for each participant group, with identical dimensions across the pair of rooms. Each set of rooms contained the same objects, but arranged in two different layouts. For blindfolded sighted participants, the rooms (length = 3.65 m, width = 1.82 m) included a TV screen, a wooden stool, two chairs, and a table. Figure 1 depicts one of these two rooms with one of the layouts used for the blindfolded sighted group. For blind participants, the rooms (length = 3.96 m, width = 2.43 m) contained two chairs, a table, a drawer box, and a small baby chair. Figure 2 depicts one of these two rooms with one of the layouts used for the blind group. The objects were selected to serve primarily as spatial landmarks for building a cognitive map, based on their semantic identity and location, rather than as large acoustic modifiers capable of altering reverberant properties. The rationale was that the task aimed to investigate whether spatialized audio could facilitate the formation of more accurate mental representations of the environment, rather than to assess how large structures influence reverberation. The use of two different rooms was motivated by practical and safety considerations: each space was adapted to the specific needs of the participant group to ensure comfortable exploration and to avoid potential risks. Although the layouts differed slightly, both rooms provided comparable opportunities for participants to localize, encode, and remember object positions. This approach maintained ecological validity by simulating realistic household environments while ensuring accessibility for all participants.

As a part of the experiment, a photograph of each room was captured using SnapStick (Shafique et al., 2025), a smartphone application designed for scene description for blind individuals. The image was processed to generate an auditory representation of the spatial layout, which was then converted into binaural audio using the 3D Tune-In Toolkit (Cuevas-Rodŕıguez et al., 2019). Binaural rendering was performed with a KEMAR dummy head from the SONICOM dataset (Engel et al., 2023) and a BRIR-based reverberation model (Engel et al., 2021) simulating room acoustics similar to the test environment. The audio was delivered through Sony MDR7506 headphones, with a Supperware head tracker continuously updating the virtual source positions according to head movements.

Each participant completed the experiment under two auditory feedback conditions: non-spatialized and binaural. In the non-spatialized condition, participants received a descriptive verbal audio representation of the room delivered without spatial cues. In the binaural condition, the same verbal description was rendered with spatialization to provide 3D localization. For each group, two different room layouts were prepared using the same set of objects arranged differently. To minimize learning or carryover effects, both the feedback conditions and the room layouts were randomized across participants. For example, if a participant first completed the task with the binaural condition in layout A and, after a short break, performed the task with the non-spatialized condition in the layout B, with each condition conducted in separate rooms.

At the start of the experiment, participants were seated in a designated chair serving as the reference point for spatial orientation. They first listened to the descriptive audio to obtain an initial idea of the room layout. This was followed by an active exploration phase, during which participants moved freely to assess distances between objects and the reference point. Blind participants were not allowed to use mobility aids (e.g., canes or assistive devices), as the task aimed to evaluate spatial representations formed through auditory input combined with free exploration.

**Figure 3:**
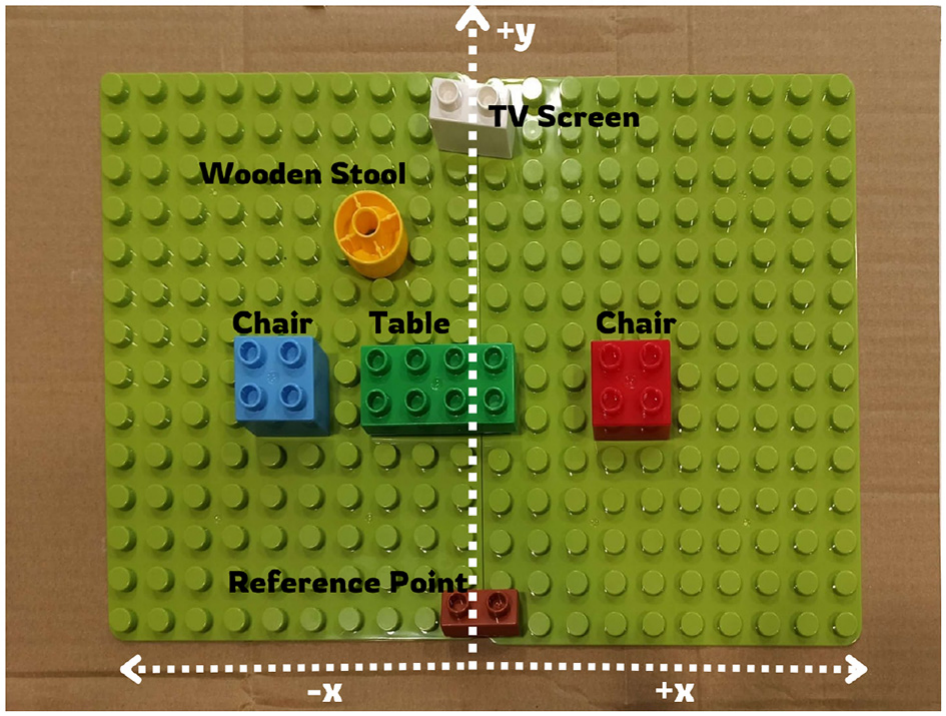
Reconstruction of the room in Figure 1 using LEGO by one of the blindfolded participants.

After exploration, participants returned to the reference point and were given a LEGO board. A reference LEGO piece was pre-placed to represent the chair they had initially occupied. Participants then reconstructed the perceived room layout by positioning LEGO pieces to represent the objects encountered during exploration. The LEGO reconstruction served as an externalization of their mental representation, effectively providing a “picture” of how each participant organized and understood the space. Thus, the task was not intended to replicate real-world navigation directly but rather to offer a tangible and comparable measure of participants' internal spatial maps formed through auditory input and exploration. An example of a participant's reconstruction is shown in Figure 3.

Once the participants had finalized their LEGO-based reconstructions, measurements were taken to assess the accuracy of their spatial perception. Specifically, the distances along the x-axis (width of room) and y-axis (length of room) between the reference LEGO and each object's LEGO representation were recorded. To ensure comparability, both distances were normalized to maintain a consistent scale. The coordinate system was defined such that the right-hand side of the reference point was considered the positive x-axis, while the left-hand side was the negative x-axis. Similarly, the region in front of the reference point was designated as the positive y-axis. These measurements were then compared to the actual spatial configuration of the room to evaluate the precision of participants' mental maps under both non-spatialized and binaural auditory feedback conditions. This approach enabled an objective assessment of how effectively participants were able to encode and reconstruct spatial layouts based on auditory cues alone.

### 2.3 Data analysis

To evaluate the similarity between reconstructed and actual room layout (Nakaya, 1997). This method compares two spatial configurations by aligning them through translation, rotation, and scaling, then measuring their degree of correspondence. The outcome is expressed as a correlation index (*r*^2^), which indicates how closely the reconstructed layout matches the reference map: values closer to 1 denote a stronger similarity, whereas values near 0 indicate poor correspondence. In addition to *r*^2^, a *t*-test was performed to compare the binaural and non-spatialized conditions in terms of the correlation index. All statistical analyses were conducted using RStudio 2023.03.0.

## 3 Results

**Figure 4:**
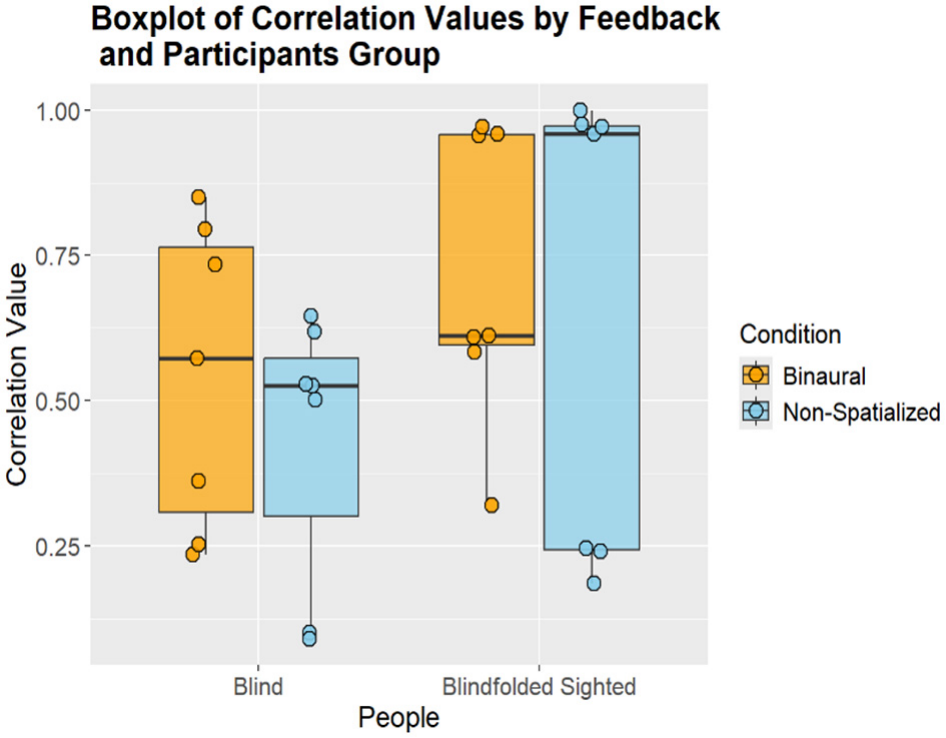
Graphical representation of correlation values of blind and sighted participants in each condition.

We assessed participants' ability to mentally map their environment using non-spatialized and binaural audio feedback. Additionally, we examined whether blind and blindfolded sighted participants differed in their capacity to interpret spatial layouts based on auditory input. For blind participants, no statistically significant differences were observed between the non-spatialized and binaural conditions. To assess the accuracy of participants' spatial reconstructions, a 2D bidimensional regression analysis was conducted, computing the correlation index between the reconstructed LEGO maps and the actual room layout. In the non-spatialized condition, the mean correlation index was *0.43* ± *0.21*, while in binaural condition, the mean correlation index was *0.54* ± *0.24*. The paired *t*-test indicated no significant differences between non-spatialized and binaural feedback in terms of correlation indices [*(t(6) = -1.51, p = 0.18*, ∣*r*∣ *= 0.71)*]. The graphical representation of the results can be seen in Figure 4.

Similarly, blind participants showed no significant differences in their ability to mentally represent object distances in the LEGO reconstruction task. The absolute distances between objects and reference point in the LEGO representations and the real environment were not significantly different under either feedback condition [non-spatialized: *t(4.46) = -0.733, p = 0.50*, ∣*r*∣ *= 0.1*; binaural: *t(6.10) = -0.344, p = 0.74*, ∣*r*∣ *= 0.3*].

Results for blindfolded sighted participants followed a similar pattern. In the non-spatialized condition, the mean correlation index was 0.65 ± 0.37. In the binaural condition, the mean correlation index was slightly higher at 0.71 ± 0.23. However, as with the blind participants, the paired *t*-test revealed no significant differences in either the correlation indices [*t(6) = -0.323, p = 0.75*, ∣*r*∣ *= -0.46*] between conditions. Figure 4 presents the graphical representation of the correlation results for the blindfolded sighted participants.

Furthermore, blindfolded-sighted participants exhibited no significant differences in object distance from the reference point in each condition. The *t*-tests showed that the absolute distances between objects and reference point in both LEGO reconstruction and actual room layout were not significantly different [non-spatialized: *t(4.10) = -0.763, p = 0.49*, ∣*r*∣ *= 0.35*; binaural: *t(4.86) = -1.356, p = 0.23*, ∣*r*∣ *= 0.1*].

It is important to note that we did not directly compare blind and sighted participants, as the experimental rooms for each group differed to accommodate their specific needs. Due to the slight differences in environmental setup, statistical comparisons were conducted separately for each group.

Overall, the findings suggest that binaural audio feedback did not confer a significant advantage in participants' ability to construct accurate mental representations of their surroundings. Both blind and blindfolded sighted participants performed similarly in both conditions, indicating that the additional spatial cues provided by binaural feedback did not substantially enhance spatial perception compared to non-spatialized feedback.

Finally, an a priori power analysis (G*Power, α *= 0.05*, power = *0.8*) indicated that ~ 34 participants per group would be required to detect a medium effect size (*Cohen's d = 0.5*). With only seven participants per group, the current study was underpowered, which may partly explain the absence of significant effects.

## 4 Discussion

This study aimed to examine how blind and blindfolded sighted individuals perceive and mentally represent their environment when using spatialized audio, comparing binaural and non-spatialized auditory feedback. Contrary to expectations based on prior work suggesting that binaural audio cues can enhance spatial awareness and navigation skills (Peterson et al., 1996; Denis et al., 2012), our results showed no significant performance differences between the two feedback conditions.

It is important to clarify that the participants received verbal descriptions of object locations, as feedback, which were presented either non-spatialized or rendered with binaural spatialization. Thus, the spatialized cues were added only to speech-based information, rather than being delivered as continuous auditory landmarks or non-verbal sonification. This may have constrained the potential benefits of binaural rendering, since participants were not continuously exposed to spatialized auditory input during exploration or reconstruction. Our findings therefore relate specifically to spatialization of descriptive audio and should not be generalized to all forms of binaural feedback.

The interpretation of these findings is further shaped by the sensory context of the task. Participants were able to freely explore the environment, relying on proprioceptive and tactile cues to complement the initial auditory description. Such redundancy in spatial information may have reduced reliance on binaural cues, thereby limiting their measurable impact (Katz et al., 2008). This interpretation is consistent with Loomis et al. (2001), who demonstrated that blind individuals frequently rely heavily on proprioceptive and vestibular feedback for navigation. In our task, proprioceptive and tactile cues alone may have provided sufficient information for participants to construct a cognitive map, reducing the added contribution of spatialized auditory input.

Another design consideration is the temporal structure of the task. Auditory descriptions were presented before the exploration and reconstruction phases, separated by a delay in which participants moved around the room. This structure likely increased demands on echoic memory, which typically retains auditory input for only a few seconds (Baddeley, 1997). While blind individuals may show enhanced auditory memory compared to sighted individuals (Arcos et al., 2022), the absence of continuous binaural cues during exploration and reconstruction could have diminished the benefits of spatialization. In such contexts, participants may have prioritized proprioceptive and tactile information acquired during exploration, overshadowing auditory contributions.

Our findings align with earlier studies that also reported limited or task-specific benefits of spatialized audio. For example, Picinali et al. (2014) examined navigation strategies in virtual auditory environments and found no consistent improvements with spatialized cues. Similarly, Katz et al. (2008) observed that spatialization did not always enhance perceptual judgments of sound localization. Although these studies focused on different domains, navigation strategies and localization judgments rather than descriptive reconstruction, they highlight that the advantages of spatialized audio are not universal and depend strongly on task demands.

One key factor that may explain the absence of binaural benefits in our study is the lack of prior training. Research has consistently shown that training enhances the effectiveness of spatialized audio. Bǎlan et al. (2015) demonstrated that both blind and sighted individuals can improve their perception of spatialized cues with targeted training, while Bastola et al. (2023) emphasized the need for tailored training programs to improve the usability of assistive technologies for blind users. Suzuki et al. (2020) similarly underscored the role of active listening and practice in improving spatial perception with binaural displays. In line with this, Ohuchi et al. (2006) showed that blind individuals can successfully navigate unfamiliar environments and form cognitive maps through practice in virtual auditory mazes. Taken together, these findings suggest that experience and structured training are critical for fully leveraging the potential of spatialized audio.

It is also necessary to address potential concerns regarding learning or carryover effects. To minimize such biases, we used different layouts with the same set of objects across the two conditions and randomized both the order of auditory feedback (binaural vs. non-spatialized) and the sequence of layouts. This design ensured that participants could not rely solely on prior knowledge of the environment when completing the second condition.

Finally, several methodological limitations must be acknowledged. First, the two rooms used for blind and blindfolded sighted participants were not identical, due to practical and safety considerations. While they were designed to be equivalent in complexity, this difference prevents direct statistical comparisons across groups. Second, the objects selected were common household items intended to provide ecologically valid landmarks rather than strong acoustic modifiers. As a result, the task primarily assessed spatial-semantic mapping and proprioception rather than perception of acoustically complex environments. Third, hearing status was assessed only through self-report rather than objective audiometric testing. Although no participant reported hearing impairments or use of hearing aids, the lack of formal verification represents a methodological limitation. Finally, the relatively small sample size, while consistent with other exploratory studies in this population, restricts the generalizability of the findings.

Our findings suggest that binaural rendering of descriptive audio alone does not confer a measurable advantage over non-spatialized feedback in supporting spatial representations, at least under conditions where participants can also rely on proprioceptive and tactile cues. This does not negate the potential of binaural audio in general but highlights the importance of task design, training, and integration with other sensory modalities. Future research should directly compare descriptive audio and non-verbal auditory feedback (e.g., sonification or continuous auditory landmarks) to better assess the specific contribution of spatialized cues to spatial representation. In addition, studies should further investigate the role of training and prior experience, as structured practice may be essential for unlocking the benefits of binaural feedback. Another important direction is the optimization of auditory assistive technologies, ensuring that their design aligns with the sensory and cognitive strategies commonly employed by blind individuals. Future work should also explore ways to reduce proprioceptive redundancy and to more effectively integrate auditory cues with other sensory modalities, thereby maximizing the ecological validity and real-world usefulness of spatialized audio systems. Finally, while our sample size was consistent with other exploratory studies in this field, larger and more diverse participant groups will be necessary to strengthen the generalizability of these findings. By addressing these factors, future studies may help to fully realize the potential of binaural audio as a tool for supporting spatial cognition and independent navigation in blind individuals.

## 5 Conclusion

Navigating unfamiliar environments without vision is inherently challenging, and auditory feedback can provide meaningful support in constructing spatial representations. In this study, we compared non-spatialized and binaural audio feedback to assess their impact on mental mapping in blind and blindfolded sighted participants. Our findings showed no significant differences between the two conditions, with both groups performing at comparable levels. These results suggest that, under the present task design, where verbal descriptions of object locations were used, adding binaural spatialization did not provide a measurable advantage over non-spatialized feedback.

Importantly, these findings should not be generalized to binaural audio in all contexts. Instead, they highlight that the benefits of spatialized audio may depend on factors such as task structure, the type of auditory cues provided, and participants' prior training or experience. Future research should explore the role of training and evaluate alternative forms of auditory feedback, including non-verbal or continuous spatial cues, to more fully determine the potential of binaural audio as a tool for supporting spatial cognition and independent navigation in blind individuals.

## Data Availability

The raw data supporting the conclusions of this article will be made available by the authors, without undue reservation.
